# Supplementary material for: HIVprotI: an integrated web based platform for prediction and design of HIV proteins inhibitors
Source: J Cheminform. 2018 Mar 9;10:12. doi: 10.1186/s13321-018-0266-y (PMC5845081; doi:10.1186/s13321-018-0266-y)
Supplement: Supplementary file 2 — Additional file 2. Source code of HIVProtI web server. [file 13321_2018_266_MOESM2_ESM.zip › HIVprotI_Source-code/hivprotI/weka-3-6-6/doc/weka/associations/Associator.html]

Associator


---


|  |  |  |
| --- | --- | --- |
| **Overview** \| **Package** \| **Class** \| **Tree** \| **Deprecated** \| **Index** \| **Help** \| **Weka's home** | |  |
| **PREV CLASS**   **NEXT CLASS** | **FRAMES**    **NO FRAMES**     **All Classes** |  |
| SUMMARY: NESTED \| FIELD \| CONSTR \| METHOD | DETAIL: FIELD \| CONSTR \| METHOD |  |


---


## weka.associations Interface Associator

**All Known Implementing Classes:**: AbstractAssociator, Apriori, FilteredAssociator, FPGrowth, GeneralizedSequentialPatterns, PredictiveApriori, SingleAssociatorEnhancer, Tertius

---

``` public interface Associator ```

---

| **Method Summary** | |
| --- | --- |
| `void` | `buildAssociations(Instances data)`             Generates an associator. |
| `Capabilities` | `getCapabilities()`             Returns the Capabilities of this associator. |

**Method Detail**

### buildAssociations

```
void buildAssociations(Instances data)
                       throws java.lang.Exception
```

:   Generates an associator. Must initialize all fields of the associator
    that are not being set via options (ie. multiple calls of buildAssociator
    must always lead to the same result). Must not change the dataset
    in any way.

    :   **Parameters:**: `data` - set of instances serving as training data **Throws:**: `java.lang.Exception` - if the associator has not been generated successfully

---


### getCapabilities

```
Capabilities getCapabilities()
```

:   Returns the Capabilities of this associator. Derived associators have to
    override this method to enable capabilities.

    :   **Returns:**: the capabilities of this object **See Also:**: `Capabilities`


---


|  |  |  |
| --- | --- | --- |
| **Overview** \| **Package** \| **Class** \| **Tree** \| **Deprecated** \| **Index** \| **Help** \| **Weka's home** | |  |
| **PREV CLASS**   **NEXT CLASS** | **FRAMES**    **NO FRAMES**     **All Classes** |  |
| SUMMARY: NESTED \| FIELD \| CONSTR \| METHOD | DETAIL: FIELD \| CONSTR \| METHOD |  |


---
